# Current understanding of ferroptosis in the progression and treatment of pancreatic cancer

**DOI:** 10.1186/s12935-021-02166-6

**Published:** 2021-09-09

**Authors:** Shi Dong, Xin Li, Wenkai Jiang, Zhou Chen, Wence Zhou

**Affiliations:** 1 The First School of Clinical Medicine, Lanzhou University, Lanzhou, China; 2 Department of General Surgery, Gansu Province, The First Hospital of Lanzhou University, No. 1, Donggang West Road, Chengguan District, Lanzhou City, 730000 China

**Keywords:** Ferroptosis, Iron accumulation, Lipid peroxidation, Pancreatic cancer

## Abstract

Pancreatic cancer is a highly malignant tumour of the digestive tract. Despite advances in treatment, its 5-year survival rate remains low, and its prognosis is the worst among all cancers; innovative therapeutic methods are needed. Ferroptosis is a form of regulatory cell death driven by iron accumulation and lipid peroxidation. Recent studies have found that ferroptosis plays an important role in the development and treatment response of tumours, particularly pancreatic cancer. This article reviews the current understanding of the mechanism of ferroptosis and ferroptosis-related treatment in pancreatic cancer.

## Introduction

Pancreatic cancer is a malignant tumour with an extremely high mortality rate. Because of its characteristic late diagnosis, high invasiveness, and distant metastasis, the 5-year survival rate of patients is less than 10% [1, 2]. Pancreatic ductal adenocarcinoma (PDAC) will become the second leading cause of cancer death in the United States by 2025 [3]. Ferroptosis is a form of iron-dependent regulatory cell death caused by excessive lipid peroxidation and subsequent plasma membrane rupture [4–6]. Studies have found that tumour cells require more iron than normal cells and are more susceptible to ferroptosis, making ferroptosis a new target to treat drug-resistant cancer [7–9]. Strategies regulating ferroptosis are a current research hot spot that influences the progression and treatment of pancreatic cancer. This article reviews the current understanding of the mechanism of ferroptosis and ferroptosis-related treatment in pancreatic cancer.

## Ferroptosis and its relationship with tumours

**Fig. 1:**
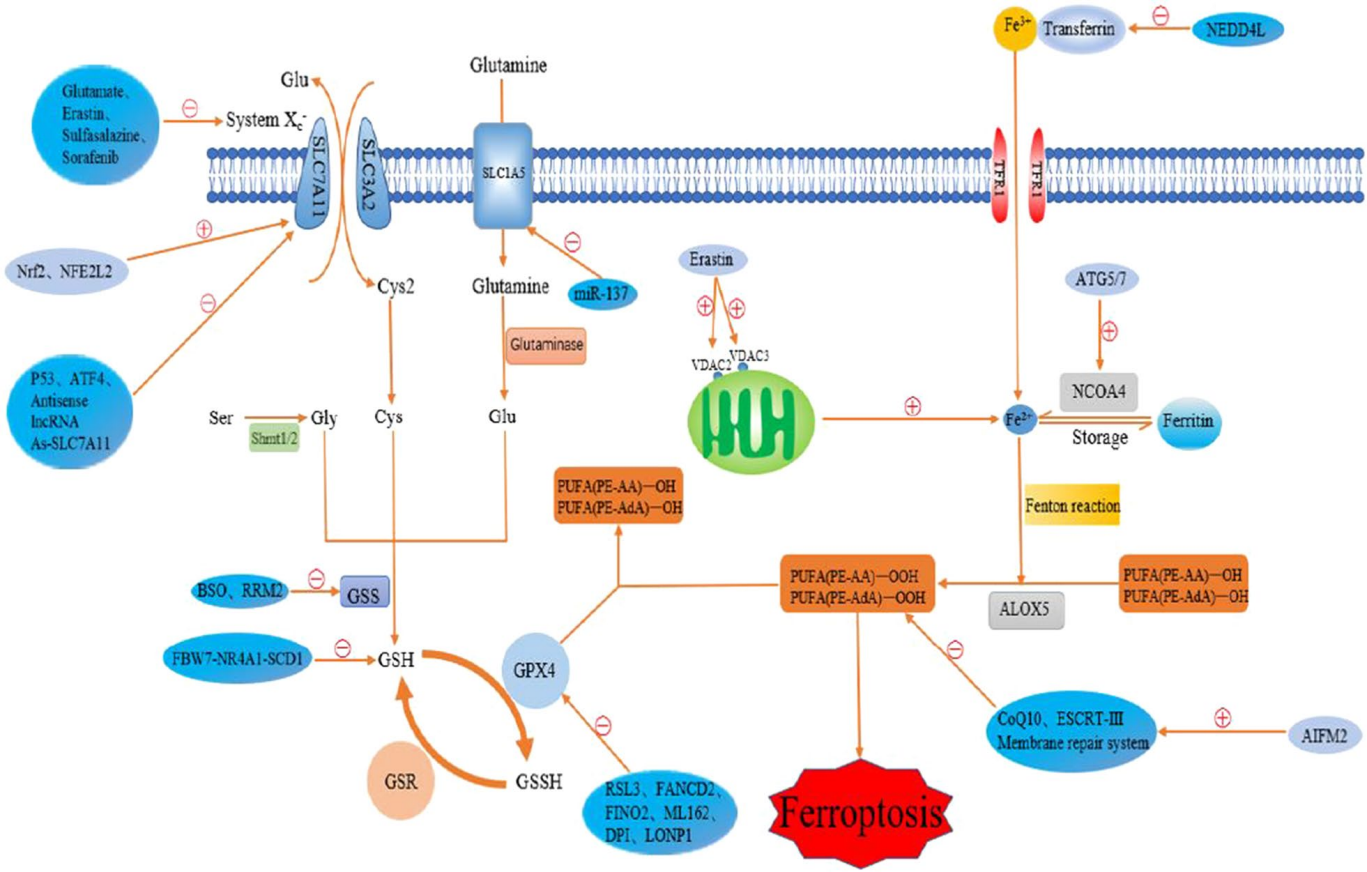
Mechanisms regulating ferroptosis. Specific mechanisms regulating ferroptosis, including various inducers and inhibitors. System X_c_^−^ transports extracellular Cys2 into the cell and transports intracellular Glu outside the cell, and then Cys2 is used to synthesize GSH. GPX4 combines with PUFA-OH to reduce reactive oxygen species generation and ultimately inhibit lipid peroxidation. Extracellular Fe^3+^ binds to the iron transporter and enters the cell through TFR1 on the cell membrane. Next, Fe^3+^ is reduced to Fe^2+^ and combined with reactive oxygen species to participate in lipid peroxidation, and finally induce ferroptosis. ⊖ indicates inhibition, and ⊕ indicates induction, *System X*_*c*_^*−*^ a glutamate/cystine antiporter; Shmt1/2, serine hydroxymethyltransferase ½, *GSS* glutathione synthase, *GSH* glutathione, *GSSH* glutathione persulfide, *GPX4* glutathione peroxidase 4, *GSR* glutathione reductase, *ALOX5* arachidonate lipoxygenase 5, *NCOA4* nuclear receptor co-activator 4, *SLC7A11* solute carrier family 7 member 11, *SLC3A2* solute carrier family 3 member 2, *SLC1A5* solute carrier family 1 member 5, *TFR1 *transferrin receptor 1, *VDAC2/3* voltage-dependent anion channel 2/3, *ATG5/7* autophagy-related protein 5/7, *Nrf2* nuclear factor E2-related factor 2, *NFE2L2* nuclear factor, erythroid 2-like 2, *ATF4* activating transcription factor 4, *lncRNA* long noncoding RNAs, *miRNA* microRNA, *PUFA* polyunsaturated fatty acid, *BSO* buthionine sulfoximine, *RRM2* ribonucleotide reductase M2, *FBW7* F-box and WD repeat domain-containing 7, *NR4A1* nuclear receptor subfamily 4 group A member 1, *SCD1* stearoyl-CoA desaturase 1, *RSL3* Ras-selective lethal 3, *FANCD2* Fanconi anaemia complementation group D2, *FINO2* 1,2-dioxolane, *ML162* (S)-enantiomer, *DPI* diphenylene iodonium, *LONP1* mitochondrial Lon protease 1, *CoQ10* coenzyme Q10, *ESCRT* endosomal sorting complex required for transport, *AIFM2* apoptosis-inducing factor mitochondria-associated 2

Ferroptosis is a non-apoptotic form of cell death. The concept of ferroptosis was first proposed by Dixon et al. in 2012 [10]. In contrast to apoptosis, autophagy and various forms of necrosis [10–12], ferroptosis is the result of iron accumulation, lipid reactive oxygen species (ROS) generation, and decreased glutathione peroxidase 4 (GPX4) activity [12]. The core of ferroptosis is cell damage caused by iron accumulation and lipid peroxidation [13, 14]. Regarding morphology, ferroptotic cells have characteristic mitochondrial atrophy, increased mitochondrial membrane density and disrupted mitochondrial membrane integrity [10, 14]. Ferroptosis is regulated by inducers and inhibitors. The inducers mainly include erastin [10], P53 [15], Ras-selective lethal 3 (RSL3) [16] and activating transcription factor 4 (ATF4) [17]. Inhibitors include buthionine sulfoximine (BSO) [18], nuclear factor, erythroid 2-like 2 (NFE2L2) [19], ferrostatin-1 (Fer-1) [10] and microRNA-137(miR-137) [20]. The mechanism of ferroptosis regulation is shown in Fig. 1. Ferroptosis is widely involved in various physiological activities of the human body, and plays an important role in the occurrence and development of diseases such as cancer, neurodegenerative diseases, and renal failure [9, 21, 22]. A summary of studies on the difference between ferroptosis and other types of cell death is shown in Table 1 [23–26].

**Table 1:** Summary of studies on the difference between ferroptosis and other types of cell death

Cell death	Ferroptosis	Apoptosis	Necroptosis	Pyroptosis	Autophagy	Necrosis
Characteristic	Programmed	Programmed	Programmed	Programmed	Programmed	Non programmed
Morphological features	Mitochondrial atrophy, increased mitochondrial membrane density and outer mitochondrial membrane rupture	Cell shrinkage, intact cell membrane and organelle structure, nuclear break	Cell volume increases and deformation, cell membrane rupture, and organelle deformation	Cell volume increase, cell membrane rupture, chromatin shrinkage	Vacuoles production, intact cell membrane, autophagosomes and phagocytic organelle formation	Cell swelling, cell membrane rupture, deformation or swelling of organelles
Biochemical features	Inhibition of system Xc-/GSH/GPX4 axis, iron accumulation, ROS production and lipid peroxidation	Oligonucleosomal DNA fragmentation	Phosphorylated MLKL translocates to the inside of the cell membrane and destroys the integrity of the cell membrane	Activation of caspase and gasdermin and release of pro-inflammatory factors	Autophagosome formation and increased lysosome activity	Reduced ATP levels Activation of RIP1, RIP3, PARP1
Regulatory pathways	system Xc-/GSH/GPX4 axis, ATG5/7-NCOA4 pathway, Keap1-NRF2 pathway, P53-SAT1-ALOX15 pathway	Caspase, P53, Bcl-2 mediated signalling pathway, death receptor pathway, mitochondrial pathway, endoplasmic reticulum pathway	TNF-α/TNFR1/RIPK1/RIPK3/MLKL	caspase-1, IL-18, IL-1β, GSDMD	mTOR, RAS, MAPK-ERK-BCL2 signalling pathway	RIP1/RIP3-MLKL related signalling pathways, PKC/MAPK/AP-1 related signalling pathway, ROS signalling pathway,
Inducers	Erastin, P53, RSL3, ATF4, NEDD4L	FASL, DCC, UNC5B	TNF-α, RIP3, RIP1, LPS, DNA damage, hypoxia	ZnO—NPs, Ivermectin	Valproate, carbamazepine, PINK1/2, rapamycin, arsenic trioxide, ULK1	TNF-α strong acid, strong alkali, toxic substance, high heat, radiation, PAMPS
Inhibitors	BSO, NFE2L2, RRM2, CA-1	c-IAP1, c-IAP2, XIAP, ML-IAP/Livin, hILP, NAIP, Z-VADFMK	caspase-8, TBK1, IKKε, RARγ, Necrostatin-1	Necrosulfonami-de	ATG4B, chloroquine, hydroxychloroquine, Spautin-1, Bafilomycin A1, hydroxychloroquine	Nec-1, Kongensin-A, NSA,

Main features of ferroptosis, apoptosis, autophagy, necroptosis, necrosis and pyroptosis, including the characteristics, morphological features, biochemical features, regulatory pathways, inducers and inhibitors.

Studies have found that tumour cells are more sensitive to intracellular iron than normal cells [27, 28]. Ferroptosis has been identified in several types of cancers, including gastric cancer [29], lung cancer [30], pancreatic cancer [31], breast cancer [32], melanoma [33] and brain cancer [34]. Studies have confirmed that activating ferroptosis effectively prevents tumour progression and enhances the effects of chemotherapy, radiotherapy and immunotherapy [35–37]. For example, Zhang et al. promoted the ferroptosis of gastric cancer cells by inhibiting miR-522, upregulating the expression of arachidonate lipoxygenase 15 (ALOX15) and inhibiting gastric cancer. This strategy also improved the therapeutic effect of paclitaxel and cisplatin [38]. Nie et al. found that retinoblastoma (Rb), nuclear factor erythroid 2-related factor 2 (NRF2) and metallothionein-1G (MT-1G) inhibit sorafenib-induced ferroptosis of liver cancer cells and reduce sensitivity to sorafenib [39]. To date, various drugs, such as erastin [10], sorafenib [39], sulfasalazine [40] and glutamate [41], have been developed to induce ferroptosis of tumour cells. The unique molecular mechanism of ferroptosis and its advantages in tumour therapy make it a likely target for cancer treatment.

## Mechanism of ferroptosis in the progression of pancreatic cancer

Since ferroptosis was identified as a novel form of cell death, the role of ferroptosis in cancer has been under extensive study. Studies have found that intervention of the ferroptosis process affects the growth and proliferation of pancreatic cancer cells and progression of pancreatic cancer. [42] This section summarizes the research progress on ferroptosis in pancreatic cancer.

### Iron accumulation and pancreatic cancer

Iron is necessary to maintain cell metabolism. Iron accumulation is an important source of ROS, a key player in ferroptosis, and is closely related to the growth and development of tumours [43, 44]. Wang et al. used erastin and RSL3 to treat pancreatic cancer cell lines (PANC1) and found that NEDD4-like E3 ubiquitin protein ligase (NEDD4L) expression is upregulated. Propidium iodide (PI) staining, revealed that the number of dead cells is increased using specific shRNA to knock out NEDD4L, and the colony formation assay confirmed that NEDD4L knockout enhances tumour suppression induced by erastin and RSL3. The experiment further proved that lactotransferrin (LTF) is the target of NEDD4L, and the decrease in LTF expression significantly inhibites the accumulation of iron in PANC1 cells. Additionally, the production of ROS and malondialdehyde, which is the end product of lipid peroxidation, is reduced, and cell mortality is also reduced. NEDD4L blocks iron accumulation and cell oxidative damage by mediating LTF degradation and ultimately inhibiting tumour growth [45]. As an antimalarial drug, artesunate (ART) inhibits various tumours. Relevant studies have found that pancreatic cancer cell death induced by ART is inhibited by deferoxamine (DFO), while increasing the content of transferrin (HTF) increases the degree of cell death, reflecting that ART-induced cell death depends on the level of free iron. Subsequent experiments proved that ferritin enters the lysosome by binding to NCOA4 and is degraded. At this time, the level of free iron increases, and ART activates lysosome activity by increasing the assembly of V-ATPase and promoting ferritin degradation. Induced ferroptosis plays a role in suppressing cancer [46, 47]. Autophagy-related genes play a critical role in regulating autophagy and are also closely related to ferroptosis. In one study, inhibiting the expression of the autophagy-related genes autophagy-related protein 5(AGT5) and ATG7 in vitro to prevent the death of erastin-induced pancreatic cancer cell lines (PANC1 and PANC2.03) significantly reduced the ferrous iron and malondialdehyde levels in the cell. The study further proved that nuclear receptor coactivator 4(NCOA4) is a receptor of ATG5 and ATG7 that mediates ferritin degradation and releases ferrous iron. The ATG5-ATG7-NCOA4 autophagy pathway is a new target to treat pancreatic cancer [48].

### Oxidative stress and pancreatic cancer

The activation of oxidative stress and weakening of the antioxidant barrier cause massive ROS production and subsequent lipid peroxidation, which ultimately induces ferroptosis [49, 50]. In this process, mitochondria, as the power station of cells, play an important role in cell death caused by oxidative stress and ferroptosis. Transcription factor A, mitochondrial (TFAM) is a member of the high mobility group protein family. Loss of its expression causes respiratory chain disorders, mitochondrial dysfunction and oxidative stress. Treatment of PANC1, Capan2 and pHsPDAC cells with the nucleoside analogue Zacitabine led to a decrease in TFAM expression, causing mitochondrial dysfunction in these cells, manifested by a decrease in mitochondrial DNA (mtDNA) replication number and cellular oxygen consumption, limited ATP production and an increase in ROS content, ultimately inducing cell death. Related experiments further proved that Zalcitabine induces ferroptosis by inducing oxidative mtDNA damage and mitochondrial function decline, as well as TFAM degradation and activation of the cyclic GMP-AMP synthase (CGAS)- stimulator of interferon gene 1 (STING1) pathway, thereby inhibiting the growth of pancreatic cancer [51]. Diaphanous homology 3 (DIAPH3) has different functions because of different tumour types. Related databases and clinical tissue sample analysis have found that DIAPH3 is highly expressed in pancreatic cancer and is positively correlated with tumour progression. DIAPH3 also promotes the proliferation and invasion of pancreatic cancer cells. Further mechanistic studies have shown that DIPAH3 inhibition promotes the antioxidant effect mediated by TrxR1 and GPX4, the key factors of selenium metabolism, increases the levels of peroxides and ROS, and ultimately inhibits the malignant phenotype of pancreatic cancer [52]. The magnetic field (MF) is considered to have an anti-tumour effect. Because of its low toxicity and noninvasiveness, MF can be used as an ideal anti-tumour treatment option. Studies have found that Wilms tumour, lung epithelial cancer, gastric cancer and pancreatic cancer exposed to MF are significantly inhibited compared with non-malignant tumours, and the inhibition rate is higher for the third day. Subsequent experiments have shown that MF promotes ROS, increases the NADPH levels, induces cell DNA damage and oxidative stress leading to ferroptosis, and inhibiting tumour proliferation [53]. Although this mechanism has not been verified in pancreatic cancer, it provides a new strategy for subsequent study.

### Lipid peroxidation and pancreatic cancer

Lipid peroxidation is the main characteristic of ferroptosis that is primarily induced by polyunsaturated fatty acids under the action of lipoxygenases and ROS and eventually activates ferroptosis and inhibits tumour development [54, 55]. Microsomal glutathione S-transferase 1 (MGST1) is a membrane-bound transferase that inhibits oxidative stress and apoptosis. In one study, MGST1 was upregulated in pancreatic cancer cell lines (CFPAC1 and PANC2. 03) treated with erastin and RSL3. Inhibiting the expression of MGST1 increased the level of intracellular malondialdehyde, but did not affect the intracellular iron content, indicating that MGST1 inhibits lipid peroxidation but not iron accumulation. Subsequent studies have found that MGST1 affects lipid peroxidation by regulating ALOX5, and in vivo experiments have confirmed that MGST1 inhibits the growth of xenotransplanted pancreatic cancer mouse tumours. Additionally, by analysing The Cancer Genome Atlas (TCGA) database, the overall survival rate of patients with high MGST1 expression was significantly lower than that of patients with low MGST1 expression, suggesting that MGST1 can be used as an indicator of prognosis for pancreatic cancer patients [56]. As part of the RAS family, ADP ribosylation factor 6 (ARF6) regulates the invasion, metastasis and proliferation of cancer cells, and is closely related to autophagy and immunity. Studies have pointed out that ARF6 is highly expressed in the pancreatic cancer cell lines PANC1 and MIA PaCa-2, and its knockdown promotes RSL3-induced ferroptosis. Further experiments have confirmed that ARF6 does not directly affect lipid peroxidation, but shapes the cell lipid components into a state that is easy to oxidize by regulating the level of ACSL4 protein, and finally induces lipid peroxidation and inhibits pancreatic cancer cell growth [57]. Hu et al. used erastin or RSL3 to treat pancreatic cancer cell lines (PANC1 and MIAPaCa2), which induced lipid peroxidation, pancreatic cancer cell death and low expression of pirin (PIR). Further studies have found that PIR inhibits lipid peroxidation by regulating the expression of acyl-CoA synthetase long-chain family member 4 (ACSL4) and ultimately affecting the tumours growth. PIR prevents the proliferation of pancreatic cancer by negatively regulating ferroptosis [58]. These studies reflect that lipid peroxidation plays an important role in the occurrence and development of pancreatic cancer and providing a new way to treat pancreatic cancer.

### System X_c_^−^/GSH/GPX4 axis and pancreatic cancer

Previous studies have confirmed that cysteine, glutathione (GSH) and GPX4 are the key foctors controlling ferroptosis [59], and system X_c_^−^ (a glutamate/cystine antiporter) can ingest cystine, which is ultimately used to synthesize GSH [60]. Ferroptosis is regulated by the system X_c_^−^/GSH/GPX4 axis. Mitochondrial Lon peptidase 1 (LONP1) is a functional enzyme that regulates mitochondrial function and cytological stability. In one study, erastin was used to treat the pancreatic cancer cell line PANC1, and western blot analysis showed that the protein levels of the key ferroptosis factors GPX4 and GSH were significantly decreased, but LONP1 expression was upregulated. Specific shRNAs were then generated to inhibit LONP1 expression. PANC1 cells with LONP1 interference were less sensitive to erastin than control cells, but GPX4 expression and the GSH content were increased; thus, LONP1 was concluded to induce a tumour suppressor effect by downregulating GPX4 expression and reducing the GSH content. Further experiments revealed that LONP1 negatively regulates the nuclear factor E2-related factor 2/Kelch-like ECH-associated protein 1 (Nrf2/Keap1) signalling pathway to affect tumour growth [61]. F-box and WD repeat domain-containing 7 (FBW7) is one of the most commonly mutated genes in human cancers that is expressed at low levels in pancreatic cancer. Ye et al. transfected FBW7 into pancreatic cancer cell lines (PANC1 and SW1990) and detected a decrease in the ratio of GSH to glutathione persulfide (GSSH). They found that intracellular ROS and the malondialdehyde level were increased using the DCFH-DA probe, indicating that FBW7 promotes ROS and lipid peroxidation in pancreatic cancer cells. They further found that FBW7 activates ferroptosis and ultimately inhibits the proliferation of pancreatic cancer cells by regulating nuclear receptor subfamily 4 group A member 1/stearoyl-CoA desaturase 1(NR4A1/SCD1) [62]. In another study, inhibiting GSH synthesis using BSO did not significantly reduce the viability of pancreatic cancer cells. This finding proved that a change in the GSH content alone is insufficient to cause ferroptosis. To further study the mechanism, mass spectrometry was performed, revealing that cystine was not only used to synthesize GSH but also converted into CoA, and the inhibitory system X_c_^−^ reduced the level of CoA and increased the content of Coenzyme Q10 (CoQ10), the key substance for CoA synthesis. The tumour cells showed ferroptosis phenotypes such as various lipids, drop formation, matrix rupture and necrosis. Thereafter, the tumour stoppeds growing and shrank. Ferroptosis caused by the combined use of pantothenate kinase inhibitor pantothenic acid inhibitors (PANKi) and BSO can be reversed by CoQ10 analogues, confirming that inhibiting GSH and CoA generates the initiation of ferroptosis in pancreatic cancer cells [63]. These studies show that targeting the system X_c_^−^/GSH/GPX4 axis is a potential new research direction for pancreatic cancer treatment.

## Ferroptosis and the early diagnosis, prognosis and staging of pancreatic cancer

Accurately performing the early diagnosis, prognosis and staging in pancreatic cancer will help to better treat and prevent the disease. Pancreatic cancer cells are more dependent on intracellular iron because of their high proliferation and DNA synthesis requirements [45]. The oxidation and reduction of iron promote the generation of oxygen free radicals, which can accelerate tumour growth. Therefore, serum ferritin and transferrin, which reflect the iron level, can be used as potential diagnostic biomarkers for pancreatic cancer detection [64, 65]. Jeong et al. found that knocking out transferrin receptor 1 (TFR1) significantly inhibits the proliferation of PDAC cells, proving that TFR1 is essential for the growth and tumourigenic phenotype of pancreatic cancer, and that it plays a crucial role in the early diagnosis of pancreatic cancer [68]. Ferroptosis can reflect the prognosis and tumour stage of pancreatic cancer patients, and genes related to ferroptosis can be used as biomarkers to predict the average survival time of pancreatic cancer patients. Studies have found that the solute carrier family 7 member 11 (SLC7A11) gene is overexpressed in pancreatic cancer cells, and the survival time of mice with SLC7A11 gene deletion induced by CRISPR-Cas9 is doubled compared with that of normal mice [63]. Other studies have found that the expression of the GPX4 gene in the pancreatic cancer group is upregulated compared with that in the normal group using TCGA database analysis. The overall survival analysis showed that high GPX4 expression is related to the increased survival rate of pancreatic cancer patients (high-expression GPX4 group: n = 131; low-expression GPX4 group: n = 43). These analyses indicate that GPX4 may be a prognostic marker for pancreatic cancer patients [69]. Additionally, ferroptosis is related to the pancreatic cancer stage. Studies have demonstrated that the expression levels of hepcidin and ferroportin in the tissue samples of pancreatic cancer patients undergoing radical surgery are highly expressed in pancreatic cancer tissues. Low expression of hepcidin and high expression of ferroportin were associated with a poor prognosis of patients, and the hepcidin expression level was associated with the pathological stage and vascular invasion in pancreatic cancer [70]. These results indicate that ferroptosis and related factors are closely related to the early diagnosis, prognosis, and tumour-node-metastasis (TNM) stage of pancreatic cancer, and have attracted widespread attention. Additionally, some researchers have identified 14 potentially valuable genes related to pancreatic cancer prognosis and ferroptosis using Gene Expression Omnibus (GEO) and TCGA databases and have analysed the mRNA and mRNA of these genes in pancreatic cancer tissues using clinical samples. Expression at the protein level confirmed that up-regulated prostaglandin-endoperoxide synthase 2 (PTGS2) expression and down-regulated metallothionein-1G (MT1G), tubulin epsilon 1 (TUBE1) and autophagy-related gene 4D (ATG4D) increase the risk of pancreatic cancer and lead to a poor prognosis; however, further experimental verification is warranted [72]. Ferroptosis is expected to play a crucial role in the comprehensive treatment of pancreatic cancer.

A summary of studies on the molecular markers of ferroptosis related to the diagnosis, prognosis and staging of pancreatic cancer is shown in Table 2.

**Table 2:** Summary of studies on the molecular markers of ferroptosis related to the diagnosis, prognosis and staging of pancreatic cancer

Molecular marker	Biomolecule type	Clinical relevance	References
Ferritin	Protein	Diagnosis, prognosis	[65, 66]
Ferroportin	Protein	Diagnosis, prognosis	[60, 65]
Hepcidin	Protein	Prognosis, TNM stage	[67]
Lipocalin 2	Protein	Diagnosis	[57, 65]
TFR1	Protein	Diagnosis, prognosis, TNM stage	[63, 65]
SLC7A11	DNA	Prognosis	[70]
GPX4	Protein	Prognosis	[67]
ARF6	Protein	Prognosis	[57]
SLC25A37/28	DNA	Prognosis	[71]
PARK2	DNA	Prognosis	[71]
AIM2	DNA	Prognosis	[71]
NUPR1	DNA	Prognosis	[73]
LCN2	DNA	Prognosis	[73]
MGST1	DNA	Prognosis	[56]
PTGS2	DNA	Prognosis	[72]
MT1G	DNA	Prognosis	[72]
TUBE1	DNA	Prognosis	[72]
ATG4D	DNA	Prognosis	[72]

Summary of the molecular markers of ferroptosis related to pancreatic cancer, including biomolecular types and clinical relevance. The biomolecular types mainly involve DNA, RNA and protein. The clinical relevance mainly includes prognosis, diagnosis, and TNM staging.

## Role of ferroptosis in pancreatic cancer treatment

### Ferroptosis and targeted therapy of pancreatic cancer

As the role of ferroptosis in pancreatic cancer was gradually uncovered, studies on its applications in the treatment of pancreatic cancer increased. As a stress-induced transcription factor, stress-induced nuclear protein 1 (NUPR1) can prevent ferroptosis. Liu et al. found that the lack of NUPR1 promotes ROS formation induced by erastin and RSL3, and inhibited the viability of pancreatic cancer cells. Further studies have shown that lipocalin 2 **(**LCN2), as the direct target gene of NUPR1, regulates iron levels in pancreatic cancer cells. By inhibiting the NUPR1-LCN2 pathway, iron accumulation and cell oxidative damage are reduced, promoting the growth of pancreatic cancer. Thus, targeting the NUPR1-LCN2 pathway provides a new strategy to treat pancreatic cancer [73]. Kuang et al. found that a lysosomal cysteine protease prevents the translocation of cathepsin B (CTSB) into the nucleus and causes DNA damage, thereby activating STING1 and ultimately inducing ferroptosis, providing a new way to improve the anticancer effect of ferroptosis [74]. GSH, which plays a key role in ferroptosis, is considered an important target for anticancer therapy, and targeting regulatory factors that affect GSH synthesis also provides a new direction for cancer treatment [75, 76]. Inhibiting SLC7A11 expression are reported to decrease the GSH content, thereby activating ferroptosis, and ultimately inhibiting the proliferation and survival of pancreatic cancer cells [77]. GPX4 is also a key factor in regulating ferroptosis process. Some studies have found that the autophagy inducers rapamycin and RSL3 cause the degradation of human pancreatic cancer cell GPX4 protein. Inhibiting GPX4 expression can enhance the anticancer activity of rapamycin and RSL3 in vivo or in vitro [78]. Presently, studies on the targeted therapy of ferroptosis in pancreatic cancer remain limited. Promoting ferroptosis in a high-glucose environment can kill pancreatic cancer cells more effectively. The pathway involves solute carrier family 2 member 1 (SLC2A1) promoting glucose uptake and inhibiting the expression of pyruvate dehydrogenase kinase 4 (PDK4), which induces lipid peroxidation and ultimately inhibits tumour growth; however, the specific mechanism and role of glucose and lipid metabolism in ferroptosis in pancreatic cancer remain to be further studied [79].

### Ferroptosis and immunotherapy of pancreatic cancer

The tumour microenvironment remains a hot research topic, and immunotherapy has also become a favoured option for cancer treatment. However, pancreatic cancer is not sensitive to immunotherapy [80]. Recent studies have found that immunotherapy is closely related to ferroptosis [38]. Ferroptosis induction to improve the sensitivity of pancreatic cancer to immunotherapy may become a new treatment strategy for pancreatic cancer. High infiltration of tumour-associated macrophages (TAMs) indicates a poor prognosis of pancreatic cancer, and M2-type TAMs can express immunosuppressive signals to promote the proliferation and invasion of pancreatic cancer cells [81]. Dai et al. found that iron and GPX4 regulate macrophage infiltration in the pancreatic tumour microenvironment, and an in vivo experiment showed that a high-iron diet or GPX4 deletion induces transmembrane protein 173(TMEM173) to promote TAM infiltration, leading to pancreatic intraepithelial neoplasia and an increased pancreas weight in mice [69]. Presently, studies on ferroptosis and pancreatic cancer treatment is still limited, and most rely on big data such as GEO and TCGA to speculate the ferroptosis regulatory factors related to pancreatic cancer immune cell infiltration, such as the aforementioned PTGS2 [72], MTIG [72] and keratin 6A (KRT6A) [82], collagen type V alpha 2 chain (COL5A2) [82], However, further experimental verification is needed, and the mechanism of action of strategies employing ferroptosis and immunotherapy requires further study. Studies on iron metabolism regulation and immune characteristics will provide new research ideas to treat and prevent pancreatic cancer.

### Ferroptosis and chemotherapy of pancreatic cancer

Gemcitabine is a first-line adjuvant chemotherapeutic drug for pancreatic cancer. Studies have indicated that the combined application of chemotherapeutic drugs and ferroptosis inducers helps improve the efficiency of chemotherapy [4], providing a new strategy to improve the sensitivity of pancreatic cancer to gemcitabine and enhance the efficacy of gemcitabine. Tang et al. analysed the gene expression profiles of parental pancreatic cancer cell lines and gemcitabine-resistant pancreatic cancer cell lines and found that components of system X_c_^−^, such as SLC7A11 and solute carrier family 3 member 2 (SLC3A2), are significantly upregulated in cells exposed to gemcitabine, indicating that ferroptosis inducers targeting system Xc- may be effective to treat gemcitabine-resistant pancreatic cancer [80]. In another study, erastin enhanced the cytotoxic effects of gemcitabine and cisplatin in two pancreatic cancer cell lines by inhibiting SLC7A11 expression and increasing the apoptosis rate, improving the efficacy of chemotherapy drugs [63]. Additionally, ARF6 increases the sensitivity of pancreatic cancer cells to gemcitabine by inhibiting iron metabolism and gemcitabine-related metabolic proteins including deoxycytidine kinase (DCK)/human equilibrative nucleoside transporter 1 (hENT1), but the specific mechanism requires further study [57]. Presently, few studies have investigated the link between ferroptosis and gemcitabine, but their combination is a feasible strategy for the comprehensive treatment of pancreatic cancer.

### Ferroptosis and radiotherapy of pancreatic cancer

In the multimodal treatment of pancreatic cancer, particularly to treat locally advanced or recurrent pancreatic tumours, radiotherapy remains a critical component [83]. Using reasonable intensity rays promotes the ferroptosis of tumour cells and reduce tumour growth, while using ferroptosis inhibitors reduces the curative effect of radiotherapy on tumours, indicating that ferroptosis modifiers can be used as radiosensitizers to improve the efficacy of radiotherapy without increasing the radiation dose and greatly reduce the serious side effects caused by overdose [8, 84]. Some studies have found that erastin, which induces ferroptosis, enhances the sensitivity of breast, cervical and lung cancer cells to radiation and promotes cell death [85–87]. Although current studies on ferroptosis combined with radiotherapy in pancreatic cancer are limited, they suggest that using radiotherapy combined with ferroptosis inducers provides a new method for advanced and recurrent pancreatic cancer.

A summary of studies on the role of ferroptosis in the treatment and drug resistance of pancreatic cancer is shown in Table 3.

**Table 3:** Summary of studies on the role of ferroptosis in the treatment and drug resistance of pancreatic cancer

Interventions	Mechanism of action	Application	In vitro/ in vivo	Animal model	References
NUPR1-LCN2	Reduces iron accumulation and inhibits ROS generation	Suppression	Both	athymic nude female mice	[73]
CTSB	Activates STING1 to induce ferroptosis	Suppression	Both	NOD-SCID female mice	[74]
CRISPR-Cas9	Inhibits the expression of SLC7A11 and reduces the content of GSH	Suppression	Both	female athymic mice	[77]
GPX4	Rapamycin and RSL3 enhance anticancer activity by inducing GPX4 protein degradation	Suppression	Both	-	[78]
SLC2A1	SLC2A1 promotes glucose uptake and inhibits the expression of PDK4, which induces lipid peroxidation	Suppression	Both	male C57BL/6 J mice	[79]
GPX4 depletion or high iron diet	Activates TMEM173 to promote TAM infiltration	Promotion	Both	C57BL/6 mice	[69]
Erastin	Inhibits SLC7A11 to improve sensitivity to gemcitabine and cisplatin	Suppression	Both	female athymic mice	[63]
ARF6	Inhibits iron metabolism and improves sensitivity to gemcitabine	Suppression	In vitro	-	[57]
NEDD4L	Degrades LTF to inhibit ferroptosis	Promotion	In vitro	-	[45]
LONP1 inhibitor	Activates the Nrf2/Keap1 signalling pathway and upregulates GPX4 expression	Promotion	In vitro	-	[61]

“Suppression” indicates that the intervention suppresses pancreatic cancer. “Promotion” indicates that the intervention promotes pancreatic cancer. “In vitro/in vivo” indicates whether the study was performed in vivo, in vitro, or both

## Summary and prospects

Pancreatic cancer remains one of the most difficult malignant tumours. Its characteristics such as high invasiveness and drug resistance have led to its high mortality rate; new treatment methods are needed. With the identification of ferroptosis, its unique molecular mechanism and role in tumours have gradually emerged. The ability of ferroptosis to improve chemotherapy, radiotherapy and immunotherapy is expected to provide new strategies for cancer treatment, particularly in pancreatic cancer [4]. However, studies on ferroptosis in pancreatic cancer remain in their infancy. A comprehensive analysis of ferroptosis-related mechanisms, such as the role of iron accumulation and lipid peroxidation in cell energy metabolism and autophagy, the effect of ferroptosis on pancreatic carcinoma stem cells, and the link between ferroptosis and tumour resistance and immune infiltration is critical. Additionally, future studies should focus more on the applications of ferroptosis in cancer treatment (particularly for pancreatic cancer), such as the following: how ferroptosis-related mechanisms can be targeted to treat pancreatic cancer with glucose and lipid metabolism disorders; and how ferroptosis can be induced or induce the recruitment of macrophages and other immune cells to cancer to play a role in immunotherapy; how iron metabolism affects the sensitivity of gemcitabine in the treatment of pancreatic cancer; the therapeutic effect of ferroptosis regulators combined with magnetic fields or radiation on pancreatic cancer. However, the synergistic effects of ferroptosis-regulating therapy with chemotherapy, immunotherapy and other antitumour strategies are expected to encourage new treatments for pancreatic cancer and other incurable cancers. We believe that ferroptosis and related factors have valuable research prospects in tumour treatment and prognostication.

## Data Availability

All the data are included in the article.

## Abbreviations

PDAC: Pancreatic ductal adenocarcinoma
ROS: Reactive oxygen species
GPX4: Glutathione peroxidase 4
RSL3: Ras-selective lethal 3
ATF4: Activating transcription factor 4
BSO: Buthionine sulfoximine
NFE2L2: Nuclear factor, erythroid 2-like 2
Fer-1: Ferrostatin-1
miR-137: MicroRNA-137
ALOX15: Arachidonate lipoxygenase 15
Rb: Retinoblastoma
NRF2: Nuclear factor erythroid 2-related factor 2
MT-1G: Metallothionein-1G
NEDD4L: NEDD4-like E3 ubiquitin protein ligase
LTF: Lactotransferrin
ATG5/7: Autophagy-related protein 5/7
NCOA4: Nuclear receptor coactivator 4
MGST1: Microsomal glutathione S-transferase 1
TCGA: The Cancer Genome Atlas
CGAS: Cyclic GMP-AMP synthase
mtDNA: Mitochondrial DNA
STING1/TMEM173: Stimulator of interferon response cGAMP interactor 1
TFAM: Transcription factor A, Mitochondrial
PIR: Pirin
ACSL4: Acyl-CoA synthetase long-chain family member 4
system X_c_^−^: The glutamate/cystine antiporter
GSH: Glutathione
LONP1: Mitochondrial Lon peptidase 1
Nrf2: Nuclear factor E2-related factor 2
Keap1: Kelch-like ECH-associated protein 1
FBW7: F-box and WD repeat domain-containing 7
NR4A1: Nuclear receptor subfamily 4 group A member 1
SCD1: Stearoyl-CoA desaturase 1
GSSH: Glutathione persulfide
TFR1: Transferrin receptor 1
SLC7A11: Solute carrier family 7 member 11
CTSB: Cathepsin B
TAMs: Tumour-associated macrophages
SLC3A2: Solute carrier family 3 member 2
SLC2A1: Solute carrier family 2 member 1
ARF6: ADP-ribosylation factor 6
DCK: Deoxycytidine kinase
hENT1: Human equilibrative nucleoside transporter 1
COQ10: CoenzymeQ10
MAPK: Mitogen-activated protein kinase
MLKL: Mixed lineage kinase domain like protein
mTOR: Mammalian target of rapamycin
PKC: Protein kinase C
RIP: Receptor-interacting serine/threonine kinase
SAT1: Spermidine/spermine N1-acetyltransferase 1
TFR1: Transferrin receptor 1
DIAPH3: Diaphanous homology 3
MF: Magnetic field
PTGS2: Prostaglandin-endoperoxide synthase 2
MT1G: Metallothionein-1G
TBUE1: Tubulin epsilon 1
ATG4D: Autophagy-related gene 4D
NUPR1: Nuclear protein 1
LCN2: Lipocalin 2
PDK4: Pyruvate dehydrogenase kinase 4
KRT6A: Keratin 6A
COL5A2: Collagen type V alpha 2 chain
